# Supplementary material for: Mechanistic Insight into the Early Stages of Toroidal Pore Formation by the Antimicrobial Peptide Smp24
Source: Pharmaceutics. 2023 Sep 28;15(10):2399. doi: 10.3390/pharmaceutics15102399 (PMC10610086; doi:10.3390/pharmaceutics15102399)
Supplement: Supplementary file 1 [file pharmaceutics-15-02399-s001.zip › pharmaceutics-2577200-supplementary.pdf]

Supplementary materials:

S1 - Electroporation simulation protocol

The following procedure was used to create toroidal pores in the bilayers during the MD simulations:

- A starting configuration for the simulation was generated using the gmx trjconv –dump function, containing both peptide, bilayer and solvent molecules, chosen from the previous set of simulations (standard AMP bilayer simulations with no external forces applied) at different timepoints after the peptide had reach an equilibrium within the top leaflet of the bilayer. Different starting configurations were used for each repeat simulation.
- A new npt simulation was started using the same simulation parameters as in the previous simulations, with the addition of a static electric field applied tangentially to the bilayer surface with a strength of 0.3 V/nm. This was done using the mdp option electric-field-z.
- The simulations were performed until a complete disruption of the bilayer was observed (can be evaluated visually or by calculating the water density over time within the normal bounds of the bilayer core). For the system used in this study this process took on average 7.89 ± 5.55 ns (n=15).
- The trajectories were visually analysed using VMD in order to find the first configuration/frame where a contiguous channel of water molecules could be seen crossing the bilayer and this configuration was isolated from the trajectory using the gmx trjconv –dump function.
- Using the configuration files generated in the previous step as the starting points, new, longer npt simulations were performed with the following modifications to the mdp files:
    - The strength of the electric field was reduced to 0.065 V/nm (needed to avoid the complete disruption of the bilayer, while still keeping the pore open. Optimal value is dependent on the size of the model, especially the Z direction.)
    - The XY compressibility of the system was reduced to 0 (the bilayer can still compress and decompress laterally by varying the size of the pore and setting this to 0 helps reduce the chance of the pore closing prematurely)
    - The center of mass correction interval (nstcomm) was reduced from every 100 to every 10 steps (helps reduce the coordinates drift caused by the unidirectional electric field in longer simulations)

S2 - Insertion of Smp24 in the negative bilayer

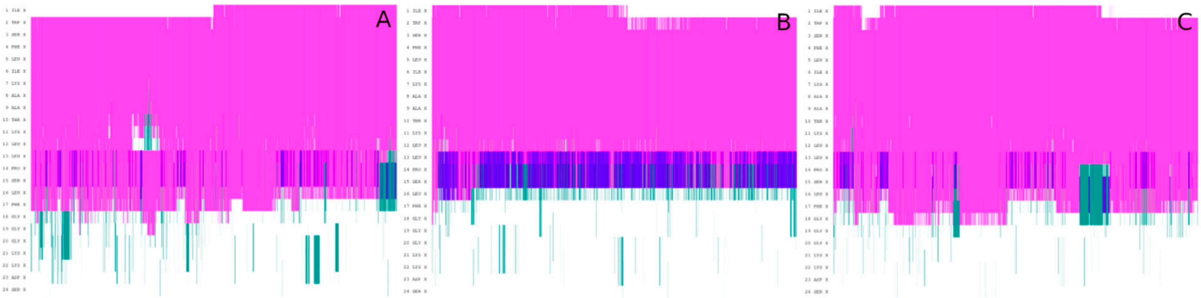

Fiugre S2.1 Secondary structure of Smp24 over time in the PCPG simulations. Pink indicates alpha-helix, blue indicates 3-10 helix, green indicates turn, yellow indicates isolated bridge and white indicates random coil.

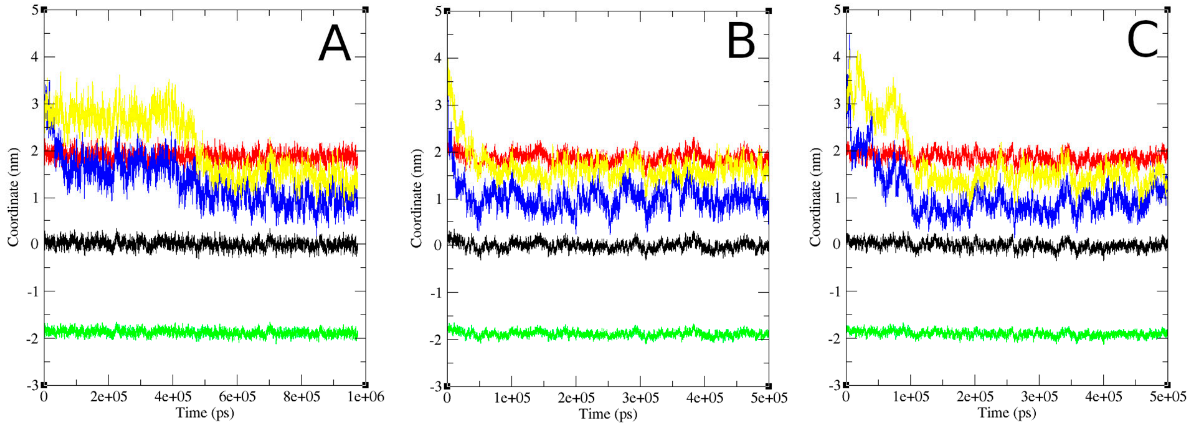

Fiugre S2.2 The centre of mass relative to the z-axes over time of select components of the PCPG Smp24 simulation. Black = centre of mass of the bilayer, Red = centre of mass of the phosphor atoms of the top bilayer leaflet, Green = centre of mass of the phosphor atoms of the bottom bilayer leaflet, Blue = centre of mass of the n-terminal, Yellow = centre of mass of the entire peptide.

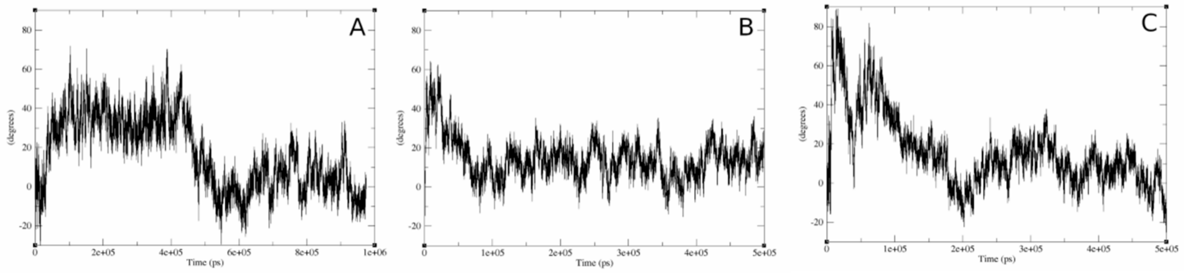

Fiugre S2.3 The angle between the primary helix (residue 1-12) and the bilayer (x-y plane) over time.

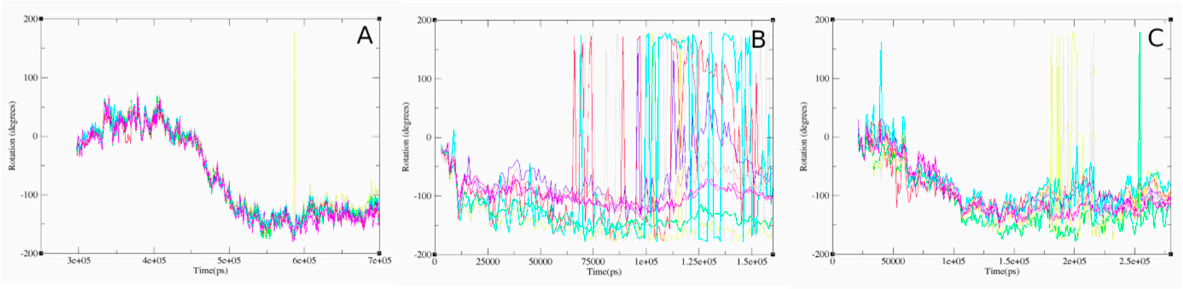

Fiugre S2.4 The relative cumulative rotation of the n-terminal end of the main alpha helix of Smp24 over time. The n-terminal inserted state taken as a starting point (0 degrees rotation). Each line represents the cumulative rotational angle over time of residues 2-10 relative to residue 1 and 11.

S3 – Inserted structure and position

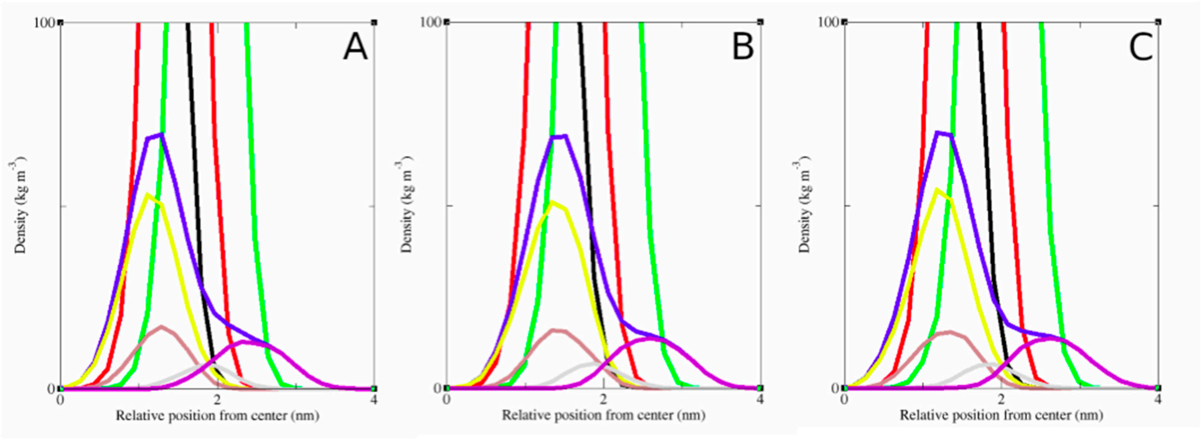

Figure S3.1 Partial density profiles of Smp24 inserted into PCPG bilayers, with positions relative to the z-axes. Black = Lipid acyl chains, Red = Lipid glycerol esters, Green = lipid headgroups with phosphates, Blue = whole peptide, Yellow = primary helix, brown = secondary helix, Grey = glycine hinge and purple = polar tail region.

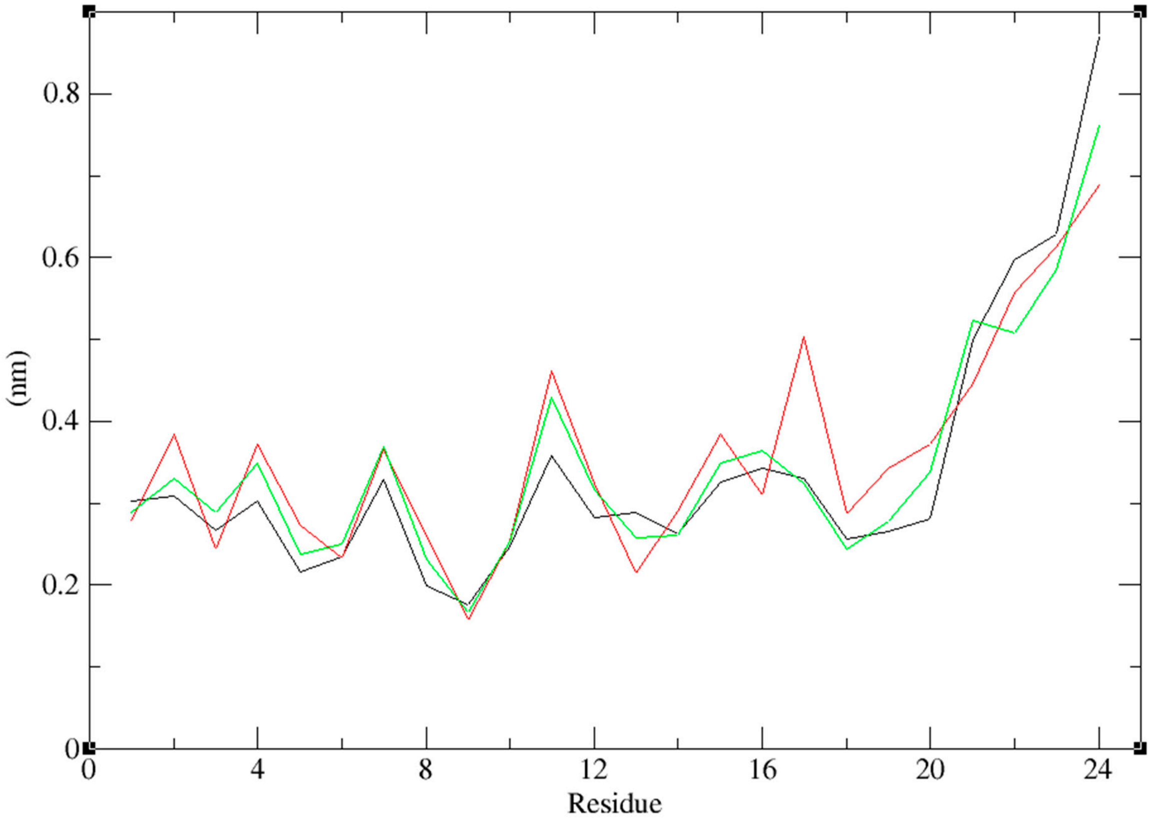

Figure S3.2 RMSF of smp24 after insertion into the DOPC:DOPG bilayers. Black = simulation A, red = simulation B, green = simulation C.

S4 - Deuterium order parameters of the multi-peptide simulations

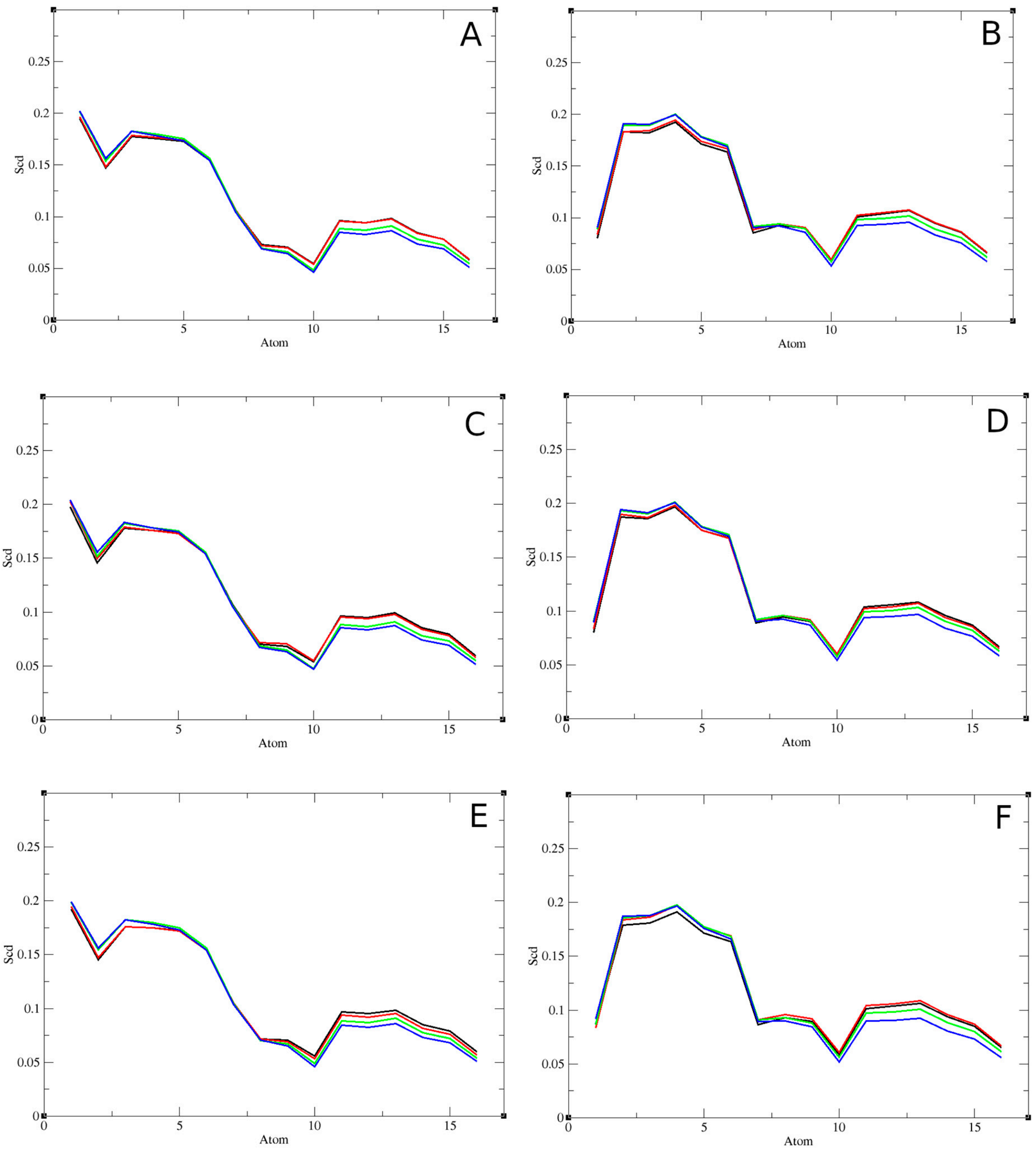

Figure S4 Deuterium order parameters of the multi-peptide simulations. A = sn1 chain for all lipids, B = sn2 chain for all lipids, C = sn1 chain for DOPC lipids only, D = sn2 chain for DOPC lipids only, E = sn1 chain for DOPG lipids only, F = sn2 chain for DOPG lipids only. Black = bilayer only, red = 144 peptide to lipid ratio, green = 48 peptide to lipid ratio, blue = 32 peptide to lipid ratio.

S5 – COM of pulled peptides

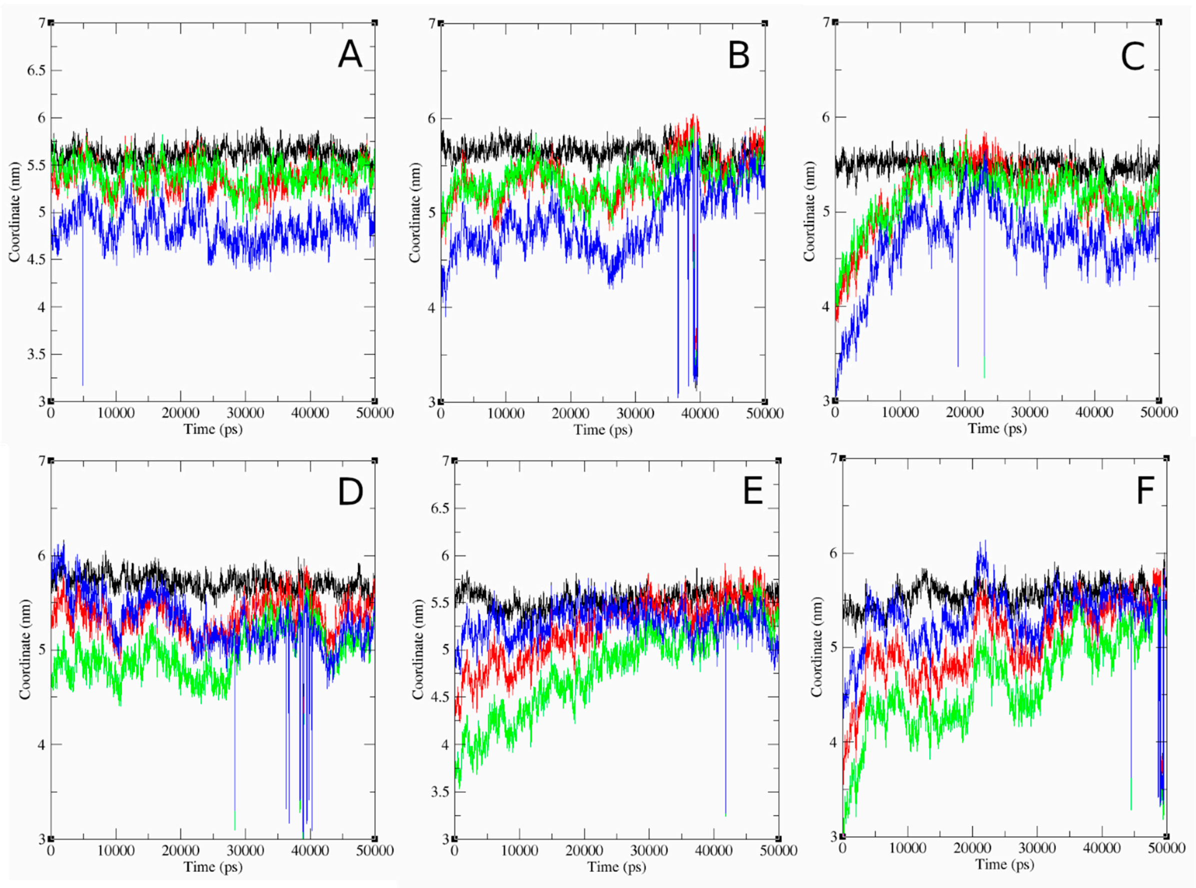

Figure S5 Center of mass over time for Smp24 after being pulled into more extreme positions within the pore. A-C) Peptide pulled from a starting position with the secondary helix oriented towards the pore opening. D-F) Peptide pulled from a starting position with the primary helix oriented towards the pore opening. Black = COM of the phosphate group of the top leaflet, red = COM of the whole peptide, green = COM of the primary helix, blue = COM of the secondary helix.

S6 Energy convergence plots

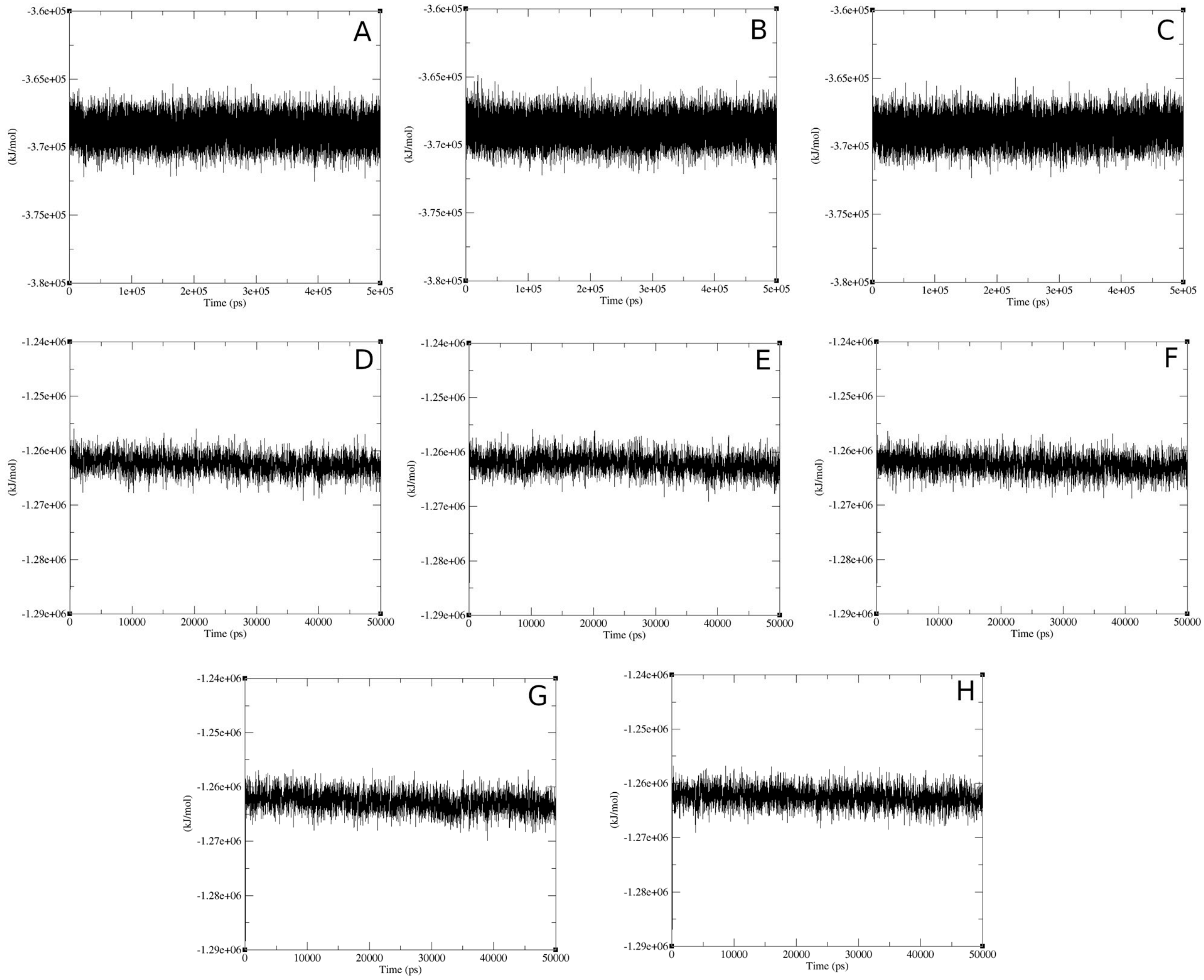

Figure S6 Energy convergence plots (potential energy of system vs time) for the Smp24 pore simulations. A-C = Single peptide pore simulations. D-H = Multipeptide pore simulations.
